# Submicron infrared spectroscopy assessment of single-cell phenotypic diversity in microbial lipid production

**DOI:** 10.1186/s12934-025-02794-x

**Published:** 2025-07-21

**Authors:** Uladzislau Blazhko, Dana Byrtusová, Volha Shapaval, Achim Kohler, Christophe Sandt, Boris Zimmermann

**Affiliations:** 1 Faculty of Science and Technology, Norwegian University of Life Sciences, Postbox 5003, Ås, 1432 Norway; 2 Synchrotron SOLEIL, Saint-Aubin, 91190 France

**Keywords:** Population heterogeneity, Single cell analysis, Infrared microscopy, Yeast, Single-cell oils

## Abstract

**Background:**

Microbial lipid production offers a sustainable method for creating biofuels, lubricants, and high-value oils, utilizing the metabolic uniqueness of diverse organisms like bacteria, yeasts, and microalgae. However, minor physicochemical variations in bioreactors, along with subtle biochemical differences in organism’s life stages, can lead to phenotypic diversity and impact the production. Therefore, monitoring, understanding and managing this diversity within bioreactors is essential in microbial biotechnology. Optical photothermal infrared (O-PTIR) spectroscopy can provide label-free chemical characterization of individual cells at sub-micron level. Here, we demonstrate the use of O-PTIR to evaluate metabolic heterogeneity within a population of oleaginous yeast *Rhodotorula graminis* in the production of free fatty acids (FFAs) and triacylglycerols (TAGs).

**Results:**

Forty yeast cells were measured by acquiring six single-point O-PTIR spectra per cell. Cell sizes were estimated from the corresponding microscopy images, while reference bulk infrared measurements of yeast biomass and pure compounds were obtained by Fourier transform infrared spectroscopies. Within the population, most of the cells have similar chemical composition, though several cells have quite different composition from the population average. Moreover, a number of cells have relatively large intra-cell chemical variability. The main chemical differences between the cells are correlated with cell sizes, and there are statistically significant size-dependent differences in cellular chemistry. Specifically, small cells have higher content of proteins than mid-size and large cells, and large cells have higher TAG-to-FFA ratio compared to mid-size cells. Characteristic wavenumbers for TAGs, FFAs and proteins can be used to estimate content of these compounds, namely 1748, 1714 and 1659 cm^− 1^ respectively.

**Conclusions:**

The O-PTIR method allows estimation of chemical composition of individual yeast cells and differentiation of two types of lipids (TAGs and FFAs). We have demonstrated that measurement at only four wavenumbers (the aforementioned wavenumbers for TAGs, FFAs and proteins plus one reference wavenumber at 1800 cm^− 1^) provides the assessment of major chemical constituents of high importance for optimization of SCO production. We foresee that rapid data acquisition through O-PTIR imaging will significantly aid in understanding and managing phenotypic diversity in microbial cells by providing a detailed representation of individual cells for population statistics.

## Background

Microbial production of lipids is gaining importance as it provides a sustainable and scalable method for producing biofuels, lubricants and high-value oils for food and feed [1–3]. Various types of microbial organisms, including bacteria, yeasts, filamentous fungi, green algae, dinoflagellates, and thraustochytrids, are employed for the production of single-cell oils (SCOs) [4]. SCO production utilizes the metabolic uniqueness of these diverse microorganisms, and thus promotes resource efficiency in the transition towards a more circular and bio-based economy. Although microbial production is conducted in bioreactors with highly controlled environments, small physicochemical heterogeneity of bioreactor environment, as well as random and subtle biochemical variations and differences in cell cycle, lead to phenotypic diversity at the single-cell level [5]. In case of SCO production, phenotypic diversity can lead to metabolic heterogeneity in the production of the desired type of lipids due to variations in gene expression, enzyme activity, substrate availability or environmental gradients (such as temperature, pH, and oxygen levels). Therefore, monitoring, understanding and managing the phenotypic diversity of microbial cells within bioreactors is a crucial aspect of microbial biotechnology. With heterogeneity data available, various strategies for reducing heterogeneity could be employed, from optimizing bioreactor design and conditions (such as, improving mixing, and pH, oxygen, temperature and nutrient availability control and stabilization), to engineering robust microbial strains with consistent gene expression and metabolic activity [6].

Currently, flow cytometry is a predominant tool employed to investigate microbial phenotypic heterogeneity in bioprocesses. In combination with fluorescent markers, this method provides rapid physical and chemical characteristics of individual cells within a population [7]. However, flow cytometry has certain limitations, such as difficulty of dealing with cell aggregates and clusters, as well as offering limited insight into complex metabolic activity and morphology of individual cells. While some of these issues can be resolved by fluorescence microscopy, in general, both flow cytometry and fluorescence microscopy are limited in determining the chemical composition of cells due to their reliance on specific fluorescence markers which may not exist for all molecules. Raman spectroscopy is a powerful, label-free technique for characterizing single cells, addressing the limitations of flow cytometry and fluorescence microscopy. For example, recent studies have demonstrated the value of coherent anti-Stokes Raman scattering (CARS) for monitoring lipid-producing yeasts [8] as well as Raman-activated cell sorting (RACS) for label-free profiling, sorting and cultivation at the single-cell level of polyphosphate-accumulating bacteria [9], aluminum-tolerant bacteria [10], and lipid-accumulating thraustochytrids [11]. Mid-infrared microspectroscopy can complement Raman-based techniques by also providing label-free chemical characterization of cells, offering detailed insights into their biochemical composition and structure [12]. Unfortunately, conventional direct-detecting infrared spectroscopy methods, such as Fourier transform infrared (FTIR) microspectroscopy have limited spatial resolution. Therefore, they are incapable of measuring individual cells of several micrometers in size, like bacteria and yeast cells.

Recent advancements in sub-micron infrared imaging techniques, such as atomic force microscopy-infrared (AFM-IR) and optical photothermal infrared (O-PTIR) spectroscopies, have revolutionized physical and life sciences by allowing chemical characterization of samples at the nanoscale [13–15]. O-PTIR spectroscopy is an advanced mid-infrared technique used for obtaining high-resolution, non-destructive chemical characterization of a variety of samples [13]. O-PTIR overcomes the diffraction limit of conventional IR microspectroscopy by measuring IR absorbance in an indirect way. In O-PTIR, the sample is placed under an optical microscope and illuminated with a tunable infrared laser (i.e. infrared pump beam). In case of absorbance of the IR radiation, the sample will warm up and thermally expand. This photothermal effect will be detected by a visible laser (i.e. visible probe beam), due to the difference in deflection of the beam before and after IR absorbance. Since spatial resolution in microscopy depends on the wavelength of the radiation used for imaging, the resolution of O-PTIR microscopy using visible light (like 532 nm green probe laser beam) is approx. 5–40 times higher than that of conventional FTIR microspectroscopy. Thus, O-PTIR allows for sub-micron spatial resolution measurement, making it a powerful tool for chemical and structural characterization in fields like biology and materials science. Recently, this technique has been demonstrated as an effective tool for assessing phenotypic heterogeneity at the single-cell level within populations of *Bacillus* strains that produce poly-3-hydroxybutyrate [16]. In that study, chemical images of bacterial cells were obtained by spectral acquisition at only two wavenumbers. Although O-PTIR spectrometers have broadband infrared laser sources with high-speed tuning, imaging at just several wavenumbers can significantly shorten acquisition time by focusing on specific spectral features of interest, rather than capturing the entire spectral range. By selectively targeting wavenumbers associated with key molecular structures via sparse wavenumber data acquisition, essential information can be measured more efficiently without the need for time-consuming scanning.

In this study, we have employed O-PTIR for assessment of single-cell phenotypic diversity in the SCO production by *Rhodotorula graminis*. *Rhodotorula* is a genus of oleaginous and carotenogenic yeasts, that can metabolize a wide range of carbon sources, and thus are of high interest in the field of biotechnology [17–22]. Regarding SCO production, *Rhodotorula* species can produce different types of lipids, including free fatty acids (FFAs) and triacylglycerols (TAGs) [23, 24]. This ability to produce both types of lipids makes them particularly valuable for industrial applications. FFAs are industrially used in the production of biodiesel, lubricants, soaps, and detergents, while TAGs are used in food and feed industry, as well as in the production of biodiesel. It is important to note that specific markers capable of distinguishing free fatty acids (FFAs) from triacylglycerols (TAGs) are currently unavailable for flow cytometry and fluorescence microscopy. Here, we demonstrate the use of O-PTIR to evaluate metabolic heterogeneity within a population of *R. graminis* in the production of FFAs and TAGs. Moreover, we assess and simulate the requirements for the sparse wavenumber data acquisition for rapid O-PTIR imaging of oleaginous yeasts.

## Materials and methods

### Microbial strain and cultivation

*Rhodotorula graminis* CCY 20-2-47 was recovered from cryopreserved stock culture (-80 °C) by inoculation on YPD agar plate (yeast extract, 10.0 g/L; peptone, 20.0 g/L; glucose 20.0 g/L; agar, 20.0 g/L) (Merck, Darmstadt, Germany) and cultivated for 72 h at 25 °C. Inoculum was prepared by transferring yeasts cells from YPD agar into 50 mL of sterile YPD broth medium (one 10 µL loop per 50 mL of fresh media; YPD media composition: yeast extract, 10.0 g/L; peptone, 20.0 g/L; glucose 20.0 g/L) (Merck, Darmstadt, Germany) in Erlenmeyer flask, and cultivated for 24 h at 22 °C under constant shaking regime (100 rpm, 50 mm 1.9 cm circular orbit) in the MAXQ 4000 incubator (Thermo Fisher Scientific, Waltham, MA, USA). To remove the residual medium after the cultivation, the inoculum cells were centrifuged (4500 rpm/5 min) and resuspended to the original volume with fresh production medium. The inoculum was added in the ratio of 1:5 (v/v) to the production medium (glucose, 52 g/L; urea, 0.45 g/L; potassium dihydrogen phosphate, 4 g/L; magnesium sulphate heptahydrate, 0.7 g/L). The cultivation in production medium was performed in Duetz Microtiter Plate System (Enzyscreen, Netherlands), which consists of 24-well extra deep microtiter plates (MTPs) with low-evaporation sandwich cowers and clamp system for mounting MTPs onto the incubator shaking platform for 96 h at 22 °C under the constant shaking regime (400 rpm and 1.9 cm circular orbit).

The collected culture broths of *Rhodotorula* fermentations were centrifuged at 3200 g for 5 min at 15 °C. The biomass was washed three times using distilled water, frozen at -80 °C, followed by freeze-drying for a minimum of 48 h in FreeZone 2.5 freeze-dryer (Labconco, USA) at -50 °C and 0.01 mbar. Details of preparation of cultivation, biomass production and production of metabolites can be found in Byrtusová et al. [22].

### Microscopy

**Fig. 1:**
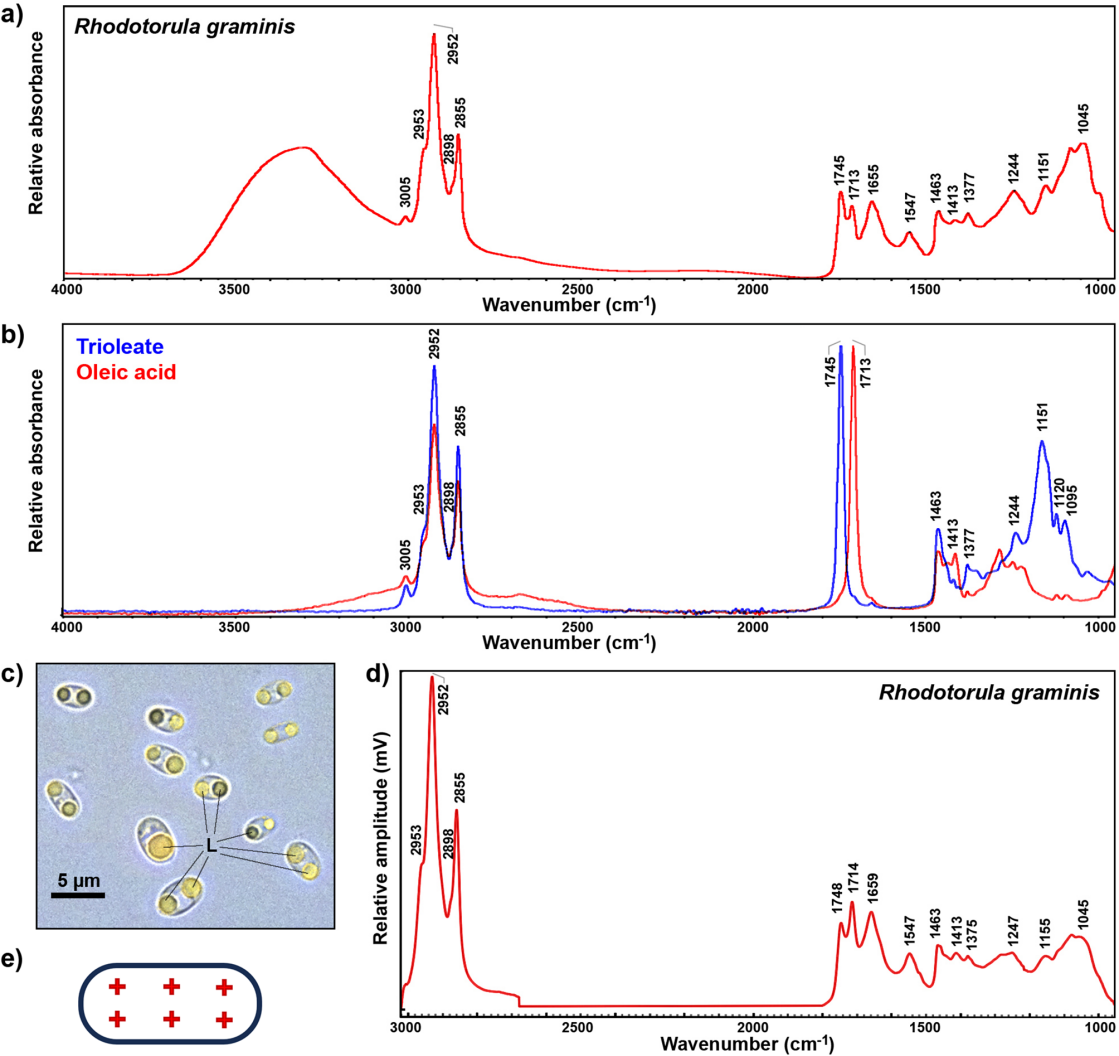
(**a**) FTIR-HTS spectrum of bulk *R. graminis* biomass. (**b**) FTIR-ATR spectra of trioleate and oleic acid. (**c**) Micrograph of *Rhodotorula* cells with lipid bodies (L). (**d**) An average spectrum of O-PTIR *R. graminis* measurements. (**e**) Schematic representation of a yeast cell with six spectral acquisition measurement points, creating 3 × 2 grid with approx. 1 μm distance between the measurement points. All spectra were baseline corrected and normalised

The micrograph (Fig. 1c) was obtained from fresh biomass on a glass microscope slide in brightfield mode with a DM6B microscope (Leica Microsystems, Wetzlar, Germany) using a 100× objective.

### Vibrational spectroscopy

O-PTIR spectra of yeast cells were measured using a mIRage infrared microscope (Photothermal Spectroscopy Corp., USA), with the infrared pump beam generated by a pulsed and tunable four-stage MIRcat mid infrared quantum cascade laser (QCL) device (DRS Daylight Solutions, USA), the probe beam generated by a continuous wave (CW) 532 nm laser, and an avalanche photodiode (APD) detector. Washed freeze-dried biomass was resuspended in distilled water with approx. concentration of 1 mg/L. Approximately 5µL of water-suspended sample was pipetted onto a barium fluoride (BaF_2_) microscope slide, and dried at room temperature for 2 h. O-PTIR spectra were acquired from individual yeast cells. Spectra were recorded with a total of 32 scans in reflection mode using a Schwarzschild 40× objective (0.78 NA), scanning over the range of 3020 − 2678 and 1801–950 cm^− 1^ (1801 − 1520, 1520 − 1185, and 1185–950 cm^− 1^), with the IR laser power within 22–77% range at a repetition (IR pulse) rate of 100 kHz, with the probe laser power within 0.06–0.18% range, and with 0.5 cm^− 1^ spectral resolution. In the aforementioned configuration of the laser and objective, the system has a focal diameter of approximately 500 nm and is capable of achieving a theoretical spatial resolution of 416 nm [25]. Six single-point spectra, creating 3 × 2 grid, were acquired in succession across each yeast cell, with approx. 1 μm distance between the measurement points. 40 individual cells were measured, with the total of 240 O-PTIR spectra. The PTIR Studio 4.3 software (Photothermal Spectroscopy Corp., USA) was used for data acquisition and instrument control.

For each measurement, a corresponding brightfield microscopy image, obtained by a digital high-resolution camera of mIRage microscope, was manually segmented using Paint.NET 5.0.13 software (Microsoft.NET Framework, USA), and further calculations of cell sizes (cross-section area) were done using Python 3.10.14 (Python Software Foundation, USA). The pixel area was calculated as a number of pixels in the cell’s mask. Then, the pixel area was converted into a metric area by multiplying it by the spatial resolution in the X and Y axes (micrometers per pixel). The spatial resolution was calculated based on the image dimensions in pixels and micrometers, which were directly obtained from the recorded measurement file.

The reference FTIR spectra of yeast biomass and reference compounds were measured with two different IR techniques using a Vertex 70 FTIR spectrometer (Bruker Optik, Germany), equipped with a globar mid-IR source and a deuterated L-alanine doped triglycene sulphate (DLaTGS) detector. The OPUS 8.2 software (Bruker Optik GmbH, Germany) was used for data acquisition and instrument control. Each sample was measured in triplicates.

The FTIR transmittance spectra (FTIR-HTS) of *Rhodotorula* biomass were measured with a high throughput screening extension (HTS-XT) unit (Bruker Optik, Germany) coupled to the Vertex 70 FTIR spectrometer. 2–3 mg of washed freeze-dried biomass was resuspended in 0.5 mL of distilled water. Approximately 8 µl of water-suspended sample was pipetted onto an IR transparent 384-well silica microplate, and dried at room temperature for 2 h. The FTIR-HTS spectra were recorded with a total of 64 scans, using Blackman-Harris 3-Term apodization, spectral resolution of 6 cm^− 1^, and digital spacing of 1.928 cm^− 1^, over the range of 4000–400 cm^− 1^, and an aperture of 5 mm. Before each sample measurement, a background (reference) spectrum was recorded using the sample-free setup (empty IR transparent microplate).

For chemical characterization of yeast chemistry, a set of model compounds was measured by FTIR spectroscopy to correlate with high positive or negative values in the principal component analyses correlation plots. Triolein (2,3-bis[[(Z)-octadec-9-enoyl]oxy]propyl (Z)-octadec-9-enoate), oleic acid ((9Z)-octadec-9-enoic acid), β-D-glucan, and gluten were purchased from Merck (Germany) and Sigma-Aldrich (USA), and used without further purification. The FTIR reflectance (FTIR-ATR) spectra of reference compounds were measured with a single reflectance-attenuated total-reflectance (SR-ATR) High-Temperature Golden Gate ATR Mk II accessory (Specac, United Kingdom) coupled to the Vertex 70 FTIR spectrometer. The FTIR-ATR spectra were recorded with a total of 32 scans, using Blackman-Harris 3-Term apodization, spectral resolution of 4 cm^− 1^, and digital spacing of 1.928 cm^− 1^, over the range of 4000–600 cm^− 1^, using the horizontal SR-ATR diamond prism with a 45° angle of incidence. Approximately 1 mg of sample was placed onto the ATR crystal for each measurement. Before the start of measurement, a background (reference) spectrum was recorded using the sample-free setup.

### Spectral preprocessing and data analysis

O-PTIR spectra were converted into second derivatives by using the Savitzky-Golay (SG) algorithm (polynomial order 2, window size 35, derivative order 2). The data were truncated to either 1780–1350 cm^− 1^ region or 1200–1000 cm^− 1^ region, and vector normalized. The spectral region of 1780–1350 cm^− 1^ was selected for the data analysis as this spectral region contains bands distinctive for lipids and proteins in oleaginous and carotenogenic yeasts [23, 26]. The spectral region of 1200–1000 cm^− 1^ predominantly has strong signals related to cell-wall carbohydrates [26]. Biochemical similarities and differences within the yeast population, based on the preprocessed O-PTIR spectral data, were estimated by using principal component analysis (PCA) on the spectral dataset truncated to 1780–1350 cm^− 1^ region.

The spectral preprocessing and the PCA was performed using Orange data mining toolbox version 3.36 (University of Ljubljana, Slovenia) [27, 28].

### Statistical analysis

The statistical analysis of the biochemical similarities and differences within the yeast population was conducted by using Pearson’s correlation coefficient (PCC) and analysis of variance (ANOVA).

**Fig. 2:**
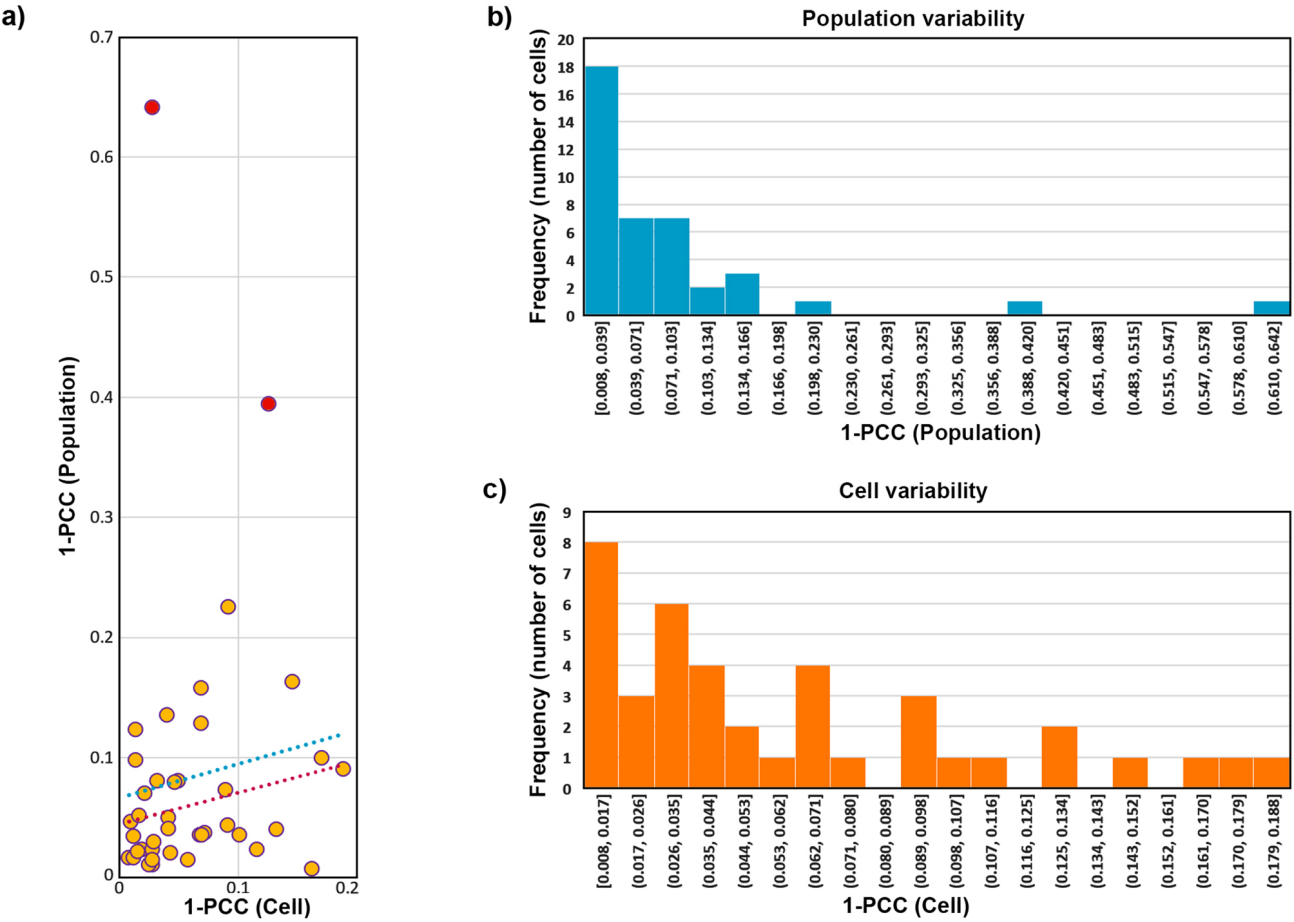
(**a**) Scatter plot with estimates of variability, based on Pearson correlation coefficient (PCC) for the dataset truncated to 1780–1350 cm^− 1^ region, within the population and within individual cells; blue dots: linear regression line (red dots: linear regression line without outliers marked with red points). (**b**) Histogram of population variability, where the variability is based on a 1-PCC value for difference between the population centroid and a cell centroid. (**c**) Histogram of cell variability, where the variability is based on an average 1-PCC value for difference between a cell centroid and a single-point spectrum (six per cell)

**Fig. 3:**
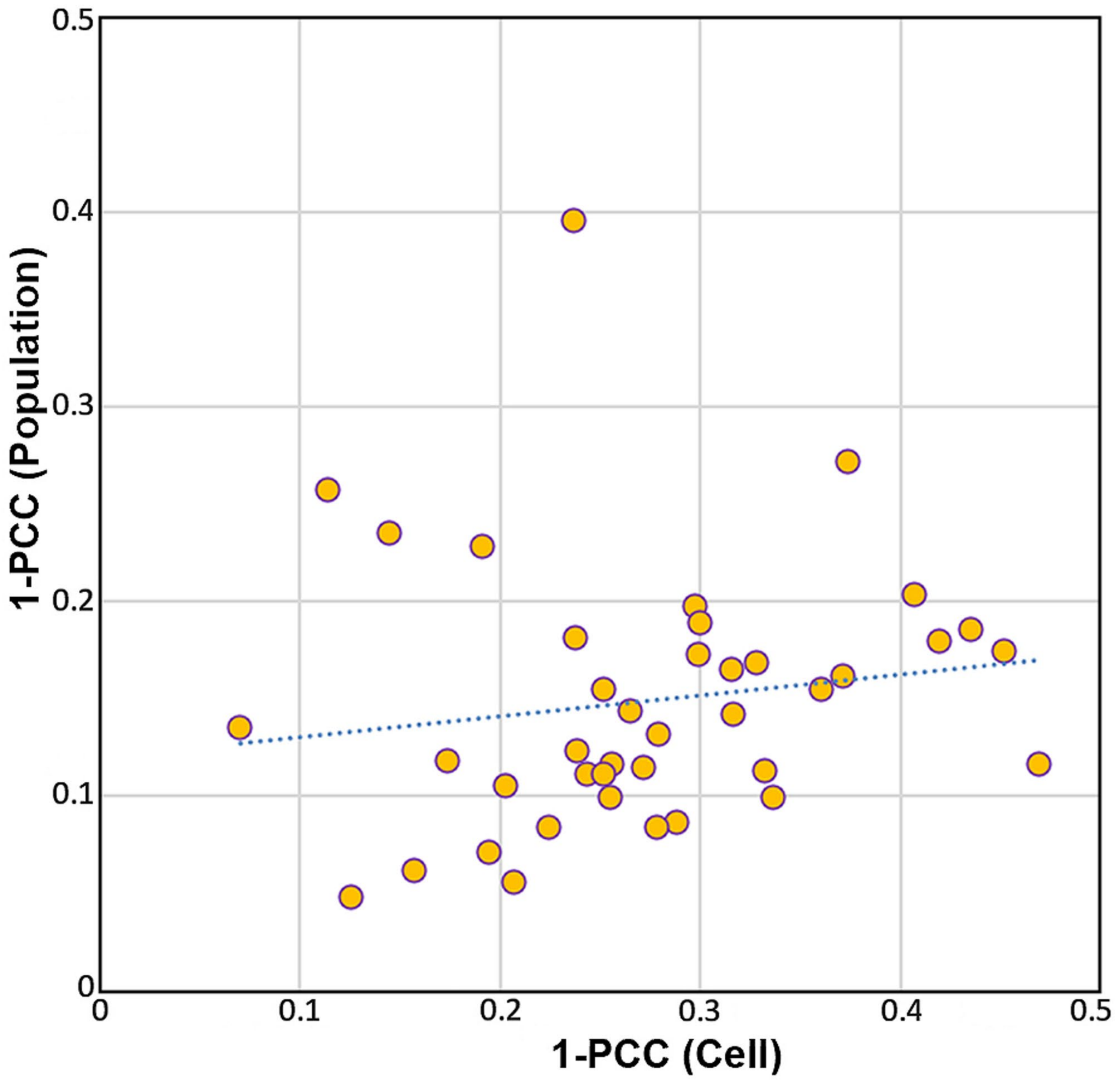
Scatter plot with estimates of variability, based on Pearson correlation coefficient (PCC) for the dataset truncated to 1200–1000 cm^− 1^ region, within the population and within individual cells; blue dots: linear regression line

The variability values of yeast chemistry within the population (i.e. inter-cell variability, assessed for 40 individual cells) and within each cell (i.e. intra-cell variability, assessed using six measurement points per cell) was calculated as 1-PCC. The PCC measures the correlation between variables, where a value of 1 indicates a perfect positive correlation. In the context of infrared spectroscopy, PCC reflects the similarity between spectral profiles, which corresponds to the similarity in the overall chemical composition as detected by IR spectroscopy. Chemical variability is quantified using Pearson distance (1-PCC), where smaller 1-PCC values indicate lower variability, and larger values reflect greater chemical differences. The PCC was calculated for two infrared regions: 1780–1350 cm^− 1^ (Fig. 2) and 1200–1000 cm^− 1^ (Fig. 3).

Intra-cell variability was estimated by calculating the average Person distance (1-PCC) from each individual spectrum to the cell centroid (i.e. centroid of all spectra belonging to the same cell, with six spectra per cell). The centroid represents the average spectrum for that specific cell. A 1-PCC value close to 0 indicates that the individual spectrum is highly similar to the cell centroid, revealing low chemical differences within the cell.

Inter-cell variability was estimated by calculating the Person distance (1-PCC) between the population centroid and a cell centroid. The population centroid was determined using all the measured O-PTIR spectra (240 spectra). Similar to intra-cell variability, a 1-PCC value close to 0 suggests that the cell centroid is very similar to the population centroid, indicating that the average chemical composition of an individual cell is very similar to the overall chemical composition of the entire microbial population. Outliers for 1-PCC values were identified based on interquartile range (IQR), with 1.5 times IQR as a multiplier for identifying outliers.

To estimate the correlation between the two sets of Person distances (calculated for the 1780–1350 cm^− 1^ and 1200–1000 cm^− 1^ spectral regions), Euclidean distances were first calculated for each set. Subsequently, the correlation between them was evaluated using the Spearman correlation method.

For the simulation of sparse wavenumber data acquisition, intensity values at four wavenumbers were taken into consideration: 1659 cm^− 1^ (as a representative frequency for proteins), 1714 cm^− 1^ (as a representative frequency for FFA), 1748 cm^− 1^ (as a representative frequency for TAGs), and at 1800 cm^− 1^ (as a representative baseline refernce frequency). Intensity values at 1800 cm^− 1^ were considered to be representative baseline reference values since this wavenumber is within the “silent” spectral region, i.e. spectral range devoid of significant chemical absorbance signals [29]. The “raw intensity” values at 1659, 1714, and 1748 cm^− 1^ were obtained by subtracting intensity values at 1800 cm^− 1^ from the coresponding three wavenumbers.

The rank-based Kruskal-Wallis test (non-parametric ANOVA alternative) was done on the ratio of intensities at 1714 and 1748 cm^− 1^, as proxies for the amount of FFAs and TAGs respectively; the intensities values had an offset (-0.1) to avoid division by zero errors for the preprocessed broadband dataset, and no offset for the simulation of sparse wavenumber data acquisition. This test was conducted instead of a standard ANOVA since the Shapiro-Wilk test for normality yielded significant results (W = 0.897, *p* < 0.001 for the preprocessed broadband dataset; W = 0.747, *p* < 0.001 for the simulation of sparse wavenumber data acquisition), indicating that the ratio values deviate significantly from normality, which violates normality assumption of the standard ANOVA test.

The PCC and ANOVA values were calculated using Python 3.10.14 (Python Software Foundation, USA).

## Results and discussion

### Spectral features in FTIR and O-PTIR spectra

The FTIR spectrum of bulk *R. graminis* biomass, shown in Fig. 1a, exhibits characteristic signals associated with TAGs and FFAs, specifically (approx. peak positions in parentheses): C–H stretching vibrations (= C–H stretching at 3005 cm^− 1^; C–H stretching in–CH_3_ and–CH_2_ at 2953, 2925, 2898 and 2855 cm^− 1^), C = O stretching in TAGs (1745 cm^− 1^), C = O stretching in FFAs (1713 cm^− 1^), CH_2_ and CH_3_ deformations (1463 and 1377 cm^− 1^), C-OH deformations (1413 cm^− 1^), and C–O stretching in–COC (1200–1000 cm^− 1^) [30–32]. Of those, the main signals of interest for this study were bands at 1745 and 1377 cm^− 1^ associated with TAGs, as well as 1710 and 1413 cm^− 1^ associated with FFAs Fig. 1b). In addition to lipid bands, the FTIR spectrum of biomass shows intensive bands associated with proteins, namely C = O stretching in amides (1655 cm^− 1^, Amide I) and CNH deformations (1547 cm^− 1^, Amide II), with phosphates, namely *P* = O stretching (approx. 1245 cm^− 1^), as well as β-glucans, namely C–C, C–O, C–O–C, CH, COH stretching, deformations and combination bands (1200–1000 cm^− 1^) [23, 32–34].

Yeasts accumulate SCOs in specialized organelles called lipid bodies, which play crucial roles in lipid metabolism by serving as dynamic storage sites for energy reserves and structural components. In the microscope image (Fig. 1c), lipid bodies are clearly visible as distinct, bright structures within *Rhodotorula* cells, and the cells themselves exhibit diverse morphologies, varying in both shape and size. The chemical assessment of this phenotypic heterogeneity was conducted by measuring 40 individual yeast cells by O-PTIR, with six measurements at different positions per cell (Fig. 1e), resulting in 240 O-PTIR spectra in total. The average O-PTIR spectrum for the whole dataset (Fig. 1d) closely resembles the FTIR spectrum of bulk *R. graminis* biomass, with all the principal IR bands being present. Although minor differences can be observed in some bands, with shifts of up to 4 cm⁻¹, high degree of similarity between the FTIR and O-PTIR spectra ensures that the spectral assignments made for the FTIR spectrum are equally applicable to the O-PTIR spectrum. It is noticeable that the relative intensities of bands in the O-PTIR spectrum are somewhat different than in the FTIR spectrum. In particular, low-wavenumber bands in the 1200–1000 cm^− 1^ spectral region are less pronounced in the O-PTIR spectrum than in the FTIR spectrum. The main spectral contribution in this region is related to β-glucans and other cell-wall carbohydrates. This difference between the two types of spectra is likely related to the physics behind how the spectral data is acquired. While in FTIR spectroscopy, the signal intensity is solely a function of IR absorbance, in O-PTIR, the signal intensity depends not only on the absorbance of IR radiation but also on the thermal expansion of the sample. In general, storage lipids such as TAGs, have higher coefficients of thermal expansion than polysaccharides, which should result with higher O-PTIR intensity values for lipids compared to polysaccharides.

### Evaluation of chemical variability

The Pearson correlation coefficient (PCC), expressed as 1-PCC, was used to assess chemical variability within the cells and within the population (Figs. 2 and 3). The results for the assessment of 1780–1350 cm^− 1^ spectral region show that, within the population, most of the cells have similar chemical composition. However, there is a tail pattern of two cells with the average chemical composition quite different from the population average (Fig. 2a and b). For most of the cells, intra-cell variability (i.e. cellular heterogeneity) is in the same value range as inter-cell variability (i.e. population heterogeneity; Fig. 2c). However, intra-cell variability is less homogenous compared to inter-cell variability, with a number of cells having relatively large cellular heterogeneity (i.e. high chemical differences within the cell). The results suggest that there is either no correlation or very weak positive correlation between inter-cell variability and cellular heterogeneity (*R*^2^ value for linear regression of 0.014 and 0.066 with and without outliers, respectively, Fig. 2a). This means that cells with relatively large cellular heterogeneity have the same or slightly higher intra-cell variability compared to the population average.

Similar to the results for the 1780–1350 cm^− 1^ spectral region, the results for the 1200–1000 cm^− 1^ spectral region show that, in general, intra-cell variability falls within the same value range as inter-cell variability (Fig. 3). Another similarity between the two spectral regions is a very weak positive correlation between inter-cell variability and cellular heterogeneity, with an R² value of 0.020 for the linear regression. A Spearman correlation of 0.219 shows a weak positive relationship between the Pearson distances calculated for the two spectral regions. This indicates that the chemical variability related to lipids and proteins (based on the spectral features in the1780-1350 cm^− 1^ spectral region) is weakly correlated with the chemical variability related to carbohydrates (based on the spectral features in the1200-1000 cm^− 1^ spectral region).

Relatively large cellular heterogeneity is expected, as the presence of large lipid bodies in oleaginous yeast cells creates distinct structural domains with unique chemistry. (Fig. 1c). Specifically, lipid bodies contain almost pure lipids [35, 36], while the cytoplasm and cell wall contain a complex mixture of proteins, nucleic acids, carbohydrates and other compounds [26]. These two distinct morphological domains each contribute with unique chemical signals to the O-PTIR spectra. Relatively large cellular heterogeneity, as detected by O-PTIR for some cells, could be the result of these cells having smaller lipid bodies, or more diverse lipid chemistry.

**Fig. 4:**
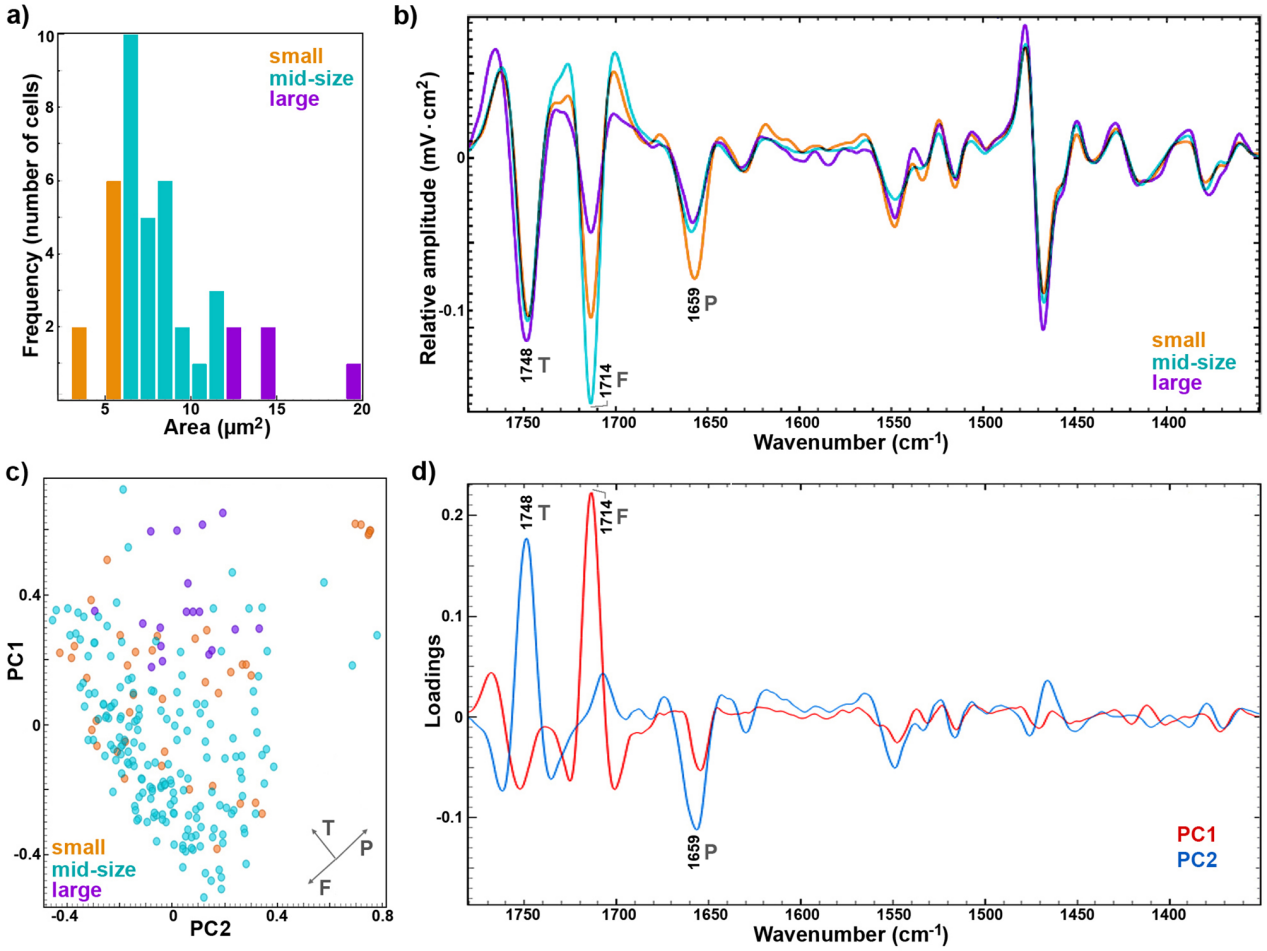
(**a**) Size distribution of measured cells (*small*: less than 6 µm^2^; *mid-size*: 6–12 µm^2^; *large*: larger than 12 µm^2^). (**b**) Average O-PTIR spectra for the three size groups (the spectral dataset was preprocessed by converting into second derivatives and vector normalized). (**c**) PCA scores with the designation of cell sizes (orange: small, blue: mid-size, purple: large); Vectors are approximating the increase in relative amount of the metabolites: TAGs (T), FFAs (F), and proteins (P). (**d**) PCA loadings on PC1 (red) and PC2 (blue); The explained variances for the first five principal components are: 28.2, 22.9, 9.9, 8.6, and 4.3%

As mentioned previously, yeast cells display diverse morphologies, with substantial variation in size (Fig. 1c). Therefore, the O-PTIR spectral dataset was partitioned into three categories according to the size of the measured cells. The cells with area smaller than 6 µm^2^ were designated as *small* cells, those with area within 6–12 µm^2^ range were designated as *mid-range* cells, and those with area larger than 12 µm^2^ were designated as *large* cells, resulting in the dataset with 8 *small*, 27 *mid-size* and 5 *large* cells (Fig. 4a). The average spectra for each size group shows that the main chemical differences between the cells are related to the relative content of TAGs, FFAs and proteins (Fig. 4b). Specifically, *small* cells have higher content of proteins than *mid-size* and *large* cells. Figure 1c shows that small cells have relatively small lipid bodies, as would be expected given their size. The main difference between *mid-size* and *large* cells is the ratio of TAGs and FFAs, where *mid-size* cells have much higher content of FFAs than *large* cells. The scores plot of the principal component analysis (PCA) of O-PTIR spectral data shows relatively extensive differences in the cellular chemistry (Fig. 4c). The loading plots corroborate that the main variance is based on lipid-to-protein ratio in the biomass (Fig. 4d), as shown by FFA-, TAG- and protein-specific signals in the first two loadings (1748, 1714 and 1659 cm^− 1^).

**Fig. 5:**
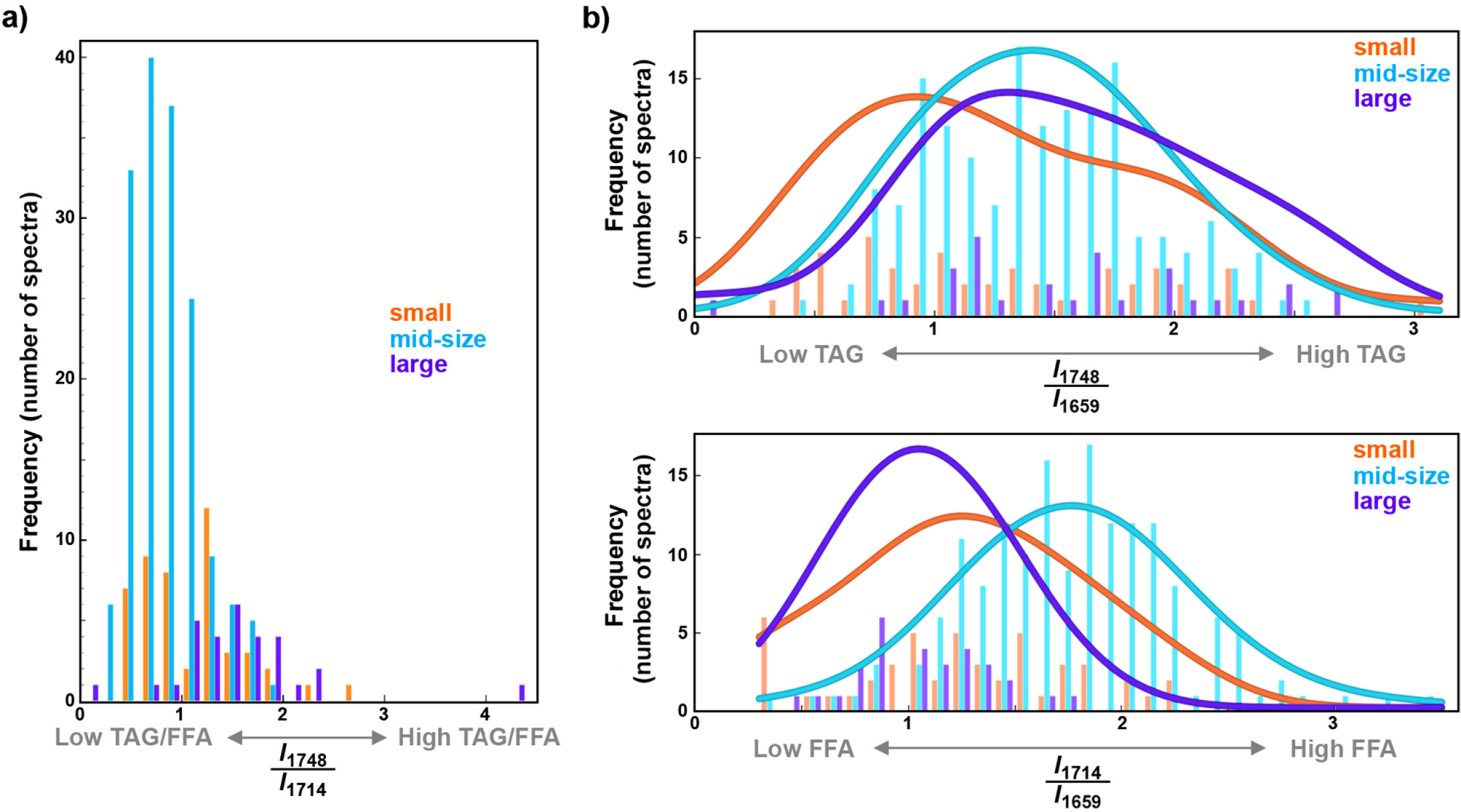
Histograms of preprocessed spectral parameters: (**a**) the ratio of intensities at 1746 cm^− 1^ and 1714 cm^− 1^ (as a proxy for ratio of triglyceride and free fatty acid content), (**b**) the ratio of intensities at 1748 cm^− 1^ and 1659 cm^− 1^ (as a proxy for triglyceride content), and (**c**) the ratio of intensities at 1714 cm^− 1^ and 1659 cm^− 1^ (as a proxy for free fatty acid content) for the three cell-sizes groups (orange: small, blue: mid-size, purple: large); the curves in (b) and (c) depict fitted kernel density estimation

The PCA results are in good agreement with the results of our previous study on lipid profiling in oleaginous yeasts by FTIR spectroscopy [23]. This previous study has shown that the intensity values at the wavenumbers related to carbonyl stretching bands of TAGs (1748 cm^− 1^), FFAs (1714 cm^− 1^), and proteins (1659 cm^− 1^) are good proxies for estimating the content of these compounds in oleaginous yeasts. Unsurprisingly, the ratio of intensities at 1748 and 1714 cm^− 1^ shows statistically significant difference among the three cell size groups (Fig. 5a; rank-based Kruskal-Wallis test: H = 45.920, *p* < 0.001). The ratio of intensities at 1714 cm^− 1^ and 1659 cm^− 1^ (as a proxy for free fatty acid content in the cell biomass) and the ratio of intensities at 1748 cm^− 1^ and 1659 cm^− 1^ (as a proxy for triglyceride content the cell biomass) also exhibit size-dependent cellular chemistry (Fig. 5b). Cells with high protein and low lipid content are overwhelmingly small. This is consistent with the results of our previous study on the monitoring of glucose fermentation by *Rhodotorula*, which showed that biomass in the early stages of cultivation has low lipid content [34]. It can be assumed that the *small* cells are likely daughter cells with similar chemical composition to the biomass at the lag phase of a fermentation. *Large*-size cells generally exhibit a high TAG content and low FFA levels, while *mid*-size cells display a markedly different lipid profile, characterized by a significantly higher FFA content relative to TAGs when compared to *large* cells.

The elevated protein content in small cells suggests elevated biosynthetic and metabolic activity. This could be indicative of active growth phases or rapid cell division, where proteins are essential for enzymatic activity, structural maintenance, and replication processes. The reduced protein content in mid-size and large cells likely reflects a shift from active growth to storage or maintenance states as cells increase in size. The difference in lipid composition between mid-size and large cells might offer further insight into their metabolic roles. The higher FFA content in mid-size cells may represent a transitional phase, potentially indicative of membrane remodelling or lipid turnover associated with adaptation to changing growth conditions [37, 38]. In contrast, the accumulation of TAGs in large cells is likely linked to their transition into stationary or post-diauxic phases with lipid bodies increasing in size from around 300 nm in late log phase to 1 μm in stationary phase [39, 40]. This observation is supported by multiple studies demonstrating that TAG accumulates during starvation and quiescence in yeast, highlighting its role as an energy reserve [36, 40]. Furthermore, TAG storage has been shown to be essential for survival under nutrient-depleted conditions [36]. The relationship between cell size and the cell cycle represents an important area of investigation. In the standard growth conditions cells, cell size may not exhibit a direct correlation with the cell cycle. However, in lipid-accumulating cells, there appears to be a notable connection between cell size and lipid metabolism, particularly under conditions of nutrient limitation (such as nitrogen limitation with excess carbon source)) or environmental stress.

**Fig. 6:**
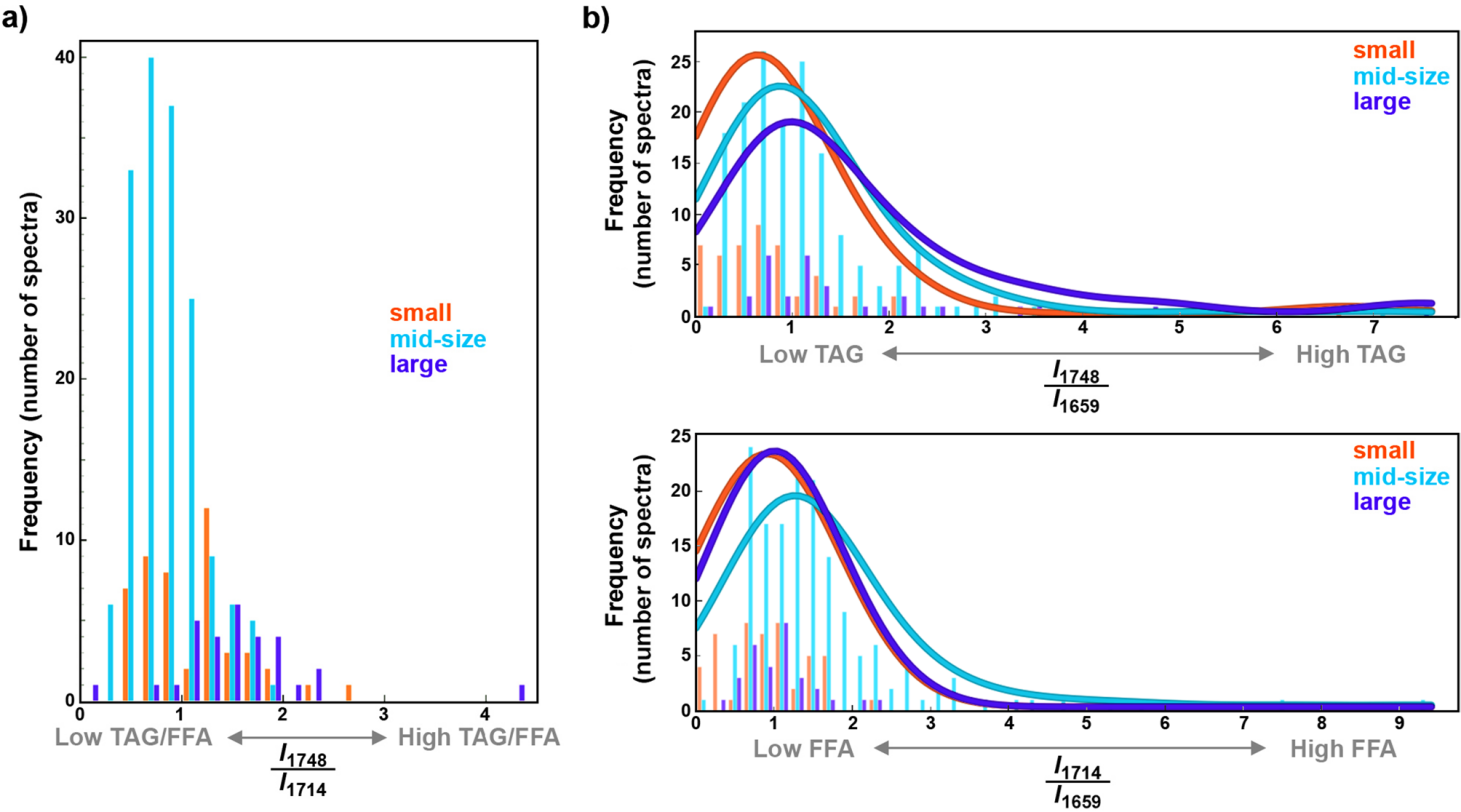
Histograms of simulation of sparse spectral acquisition at four wavelengths (1800, 1748, 1714, and 1659 cm^− 1^): (**a**) the ratio of intensities at 1746 cm^− 1^ and 1714 cm^− 1^ (as a proxy for ratio of triglyceride and free fatty acid content), (b) the ratio of intensities at 1748 cm^− 1^ and 1659 cm^− 1^ (as a proxy for triglyceride content), and (c) the ratio of intensities at 1714 cm^− 1^ and 1659 cm^− 1^ (as a proxy for free fatty acid content) for the three cell-sizes groups (orange: small, blue: mid-size, purple: large); the curves in (b) and (c) depict fitted kernel density estimation

In addition to the identification of lipid classes such as TAGs and FFAs, the assessment of lipid saturation represents a valuable parameter for comprehensive chemical characterization of microbial lipids. Studies have demonstrated that bands in the C-H stretching spectral region (3100–2800 cm⁻¹), particularly the weak = C-H stretching band around 3010 cm⁻¹, are diagnostic for quantifying lipid saturation [41, 42]. Unfortunately, the endpoint of the O-PTIR microscope used in this study is at 3020 cm⁻¹, resulting in an insufficient signal-to-noise ratio above 3000 cm⁻¹ to reliably and accurately measure this weak band. For these reasons, determination of lipid saturation was not included in the scope of this study.

### Simulation of sparse-wavelength O-PTIR measurements

For O-PTIR imaging it is crucial to identify highly specific wavenumbers for the main chemical constituents in the sample. This necessity arises because O-PTIR imaging measurements are often conducted in sparse wavenumbers mode, where images are obtained by measurement at only a few selected IR wavenumbers in order to cover a large imaging area within a reasonable acquisition time. Covering large area with hundreds of cells is critical for obtaining representative sample size for microbial population. To simulate sparse-wavelength data acquisition, we have conducted data analysis on intensity values taken at only four wavenumbers: 1659, 1714, 1748, and 1800 cm^− 1^. The intensity values at 1800 cm^− 1^ were used as a representative baseline values for correcting intensity values at the other three wavenumbers. This sparse simulation produced qualitatively very similar results (Fig. 6) to the ones obtained from the preprocessed broadband spectra (Fig. 5). Specifically, cells with high protein and low lipid content are overwhelmingly small, while large cells have in general high TAG and low FFA content. Analogous to the broadband dataset (Fig. 5a), the ratio of intensities at 1748 and 1714 cm^− 1^ shows statistically significant difference among the three cell size groups (Fig. 6a; rank-based Kruskal-Wallis test: H = 30.071, *p* < 0.001). It is important to note that sparse-mode O-PTIR imaging at the aforementioned frequencies would allow the use of a more economical device than the one used in this study. This is because only a single-stage mid-infrared quantum cascade laser device, covering the spectral range 1801–1520 cm^− 1^, would be needed, as opposed to a four-stage broadband device. Sparse-mode O-PTIR imaging, when combined with an automated bioreactor sampling, as well as automated processes for sample washing, concentration adjustment, and slide spotting (for example, using a version of the system we demonstrated [43]), could enable the characterization of phenotypic diversity within approximately 1 h of the harvesting timepoint.

## Conclusion

Here, we have investigated the use of sub-micron IR spectroscopy for microbial phenotypic heterogeneity in bioprocesses. The O-PTIR method allows estimation of chemical composition of individual yeast cells and differentiation of two types of lipids, namely triglycerides and free fatty acids. The primary chemical differences, related to protein and lipid composition and content, are associated with cell size, with statistically significant size-dependent variations in cellular chemistry (as demonstrated by the ratio of intensities at 1748 and 1714 cm⁻¹ differing significantly among the three cell size groups). Specifically, small cells have a higher protein content compared to mid-size and large cells, while large cells exhibit a higher TAG-to-FFA ratio relative to mid-size cells. We have demonstrated that measurement at only four wavenumbers provides assessment of major chemical constituents of high importance for optimization of SCO production: proteins at 1659 cm^− 1^, triglycerides at 1748 cm^− 1^, free fatty acids at 1714 cm^− 1^, and baseline reference wavenumber at 1800 cm^− 1^. This is highly important for the use of O-PTIR imaging in assessing population phenotypic heterogeneity related to the SCO production, as it allows for the relatively fast and economical acquisition of spectral data. We envision that combining automated bioreactor harvesting and sample processing with sparse-mode O-PTIR imaging and computer vision, which enables rapid data acquisition from hundreds of cells, would be invaluable in microbial biotechnology. Such measurements would provide a thorough representation of individual cells for population statistics, thereby facilitating a deeper understanding and management of the phenotypic diversity among microbial populations in industrial processes.

## Data Availability

All datasets generated for this study are available in the Zenodo repository upon reasonable request from the corresponding author: DOI 10.5281/zenodo.15097504.
